# Mimicking an Intraluminal Lesion: A Case Report on Mantle Cell Lymphoma

**DOI:** 10.7759/cureus.18870

**Published:** 2021-10-18

**Authors:** Kosisochukwu J Ezeh, Obiora Ezeudemba

**Affiliations:** 1 Internal Medicine, Jersey City Medical Center, Jersey City, USA; 2 Internal Medicine, St. Vincent Medical Center, Connecticut, USA

**Keywords:** non-hodgkin lymphoma, nhl, mantle cell lymphoma, gastritis, intra-abdominal lesion

## Abstract

Mantle cell lymphoma is typically an aggressive, rare form of non-Hodgkin lymphoma that arises from cells originating in the “mantle zone.” Here, we describe a case of mantle cell lymphoma associated with gastrointestinal bleeding, which was worked up as a possible gastrointestinal lesion or intraluminal pathology. The patient presented with symptomatic microcytic anemia associated with a positive guaiac fecal test. However, after an extensive workup to elucidate the symptomatic anemia’s etiology, mantle cell lymphoma associated with a positive guaiac test was the culprit. Usually, mantle cell lymphoma is diagnosed at a later stage involving the bone marrow and gastrointestinal tract.

## Introduction

In western countries, mantle cell lymphoma comprises approximately 3-10% of adult-onset non-Hodgkin lymphoma (NHL). Its incidence continues to rise with non-Hispanic whites and Caucasians in the United States having a higher incidence compared to other ethnicities [1]. It is a rare B-cell lymphoma defined by the translocation (11;14)(q13;q32) that results in the overexpression of cyclin D [2]. Here, we report the case of a 70-year-old caucasian male who was diagnosed with mantle cell lymphoma during a workup for a presumed intraluminal pathology.

## Case presentation

A 70-year-old male with a medical history of hypertension, osteoarthritis, constipation, and chronic back pain presented to the Emergency Department (ED) with a two-week history of dizziness. He reported feeling like his head was heavy that aggravated on standing, due to which he was afraid to ambulate with his cane. He had consulted his cardiologist a week ago and had received a prescription for aspirin with a recommendation to continue taking iron pills. He also stated that he had been anemic in the past, but was no longer taking iron pills. In the last five days, he noticed reduced oral intake and reported that he did not feel hungry. He had an episode of vomiting after meals three days before his presentation to the ED.

His last colonoscopy was performed five years ago which was unremarkable, and he was told to return in five years. He denied melena (black tarry stools), hematemesis, hematuria, nausea, fever, chills, palpitations, shortness of breath, weight changes, or consumption of other over-the-counter non-steroidal anti-inflammatory drugs such as ibuprofen, Motrin, or Advil. He endorsed that he was adherent with his medications but did not recall the names or doses of his medications.

On examination, he was afebrile with a blood pressure of 132/75 mmHg, heart rate of 95 beats per minute, respiratory rate of 16 breaths per minute, and oxygen saturation of 98% on room air. His orthostatic was positive. His skin was pale and he had conjunctiva. Complete blood count (CBC) was significant for anemia (hemoglobin 7.1 g/dL, mean corpuscle volume 66 fL), thrombocytosis (platelets 529 × 10^9^/L), and leukocytosis (white blood count 15.7 × 10^9^ cells/L, predominantly segmented 83%). The basic metabolic panel was remarkable for hypokalemia (potassium 3.2 mmol/L) and altered kidney function (blood urea nitrogen [BUN] 34 mg/dL, creatinine 1.43 mg/dL; in May 2019: BUN 20 mg/dL, creatinine 0.9 mg/dL). Serum glucose was 132 mg/dL. His liver function tests and lipid profile were within normal limits. The fecal guaiac test was positive.

**Figure 1:**
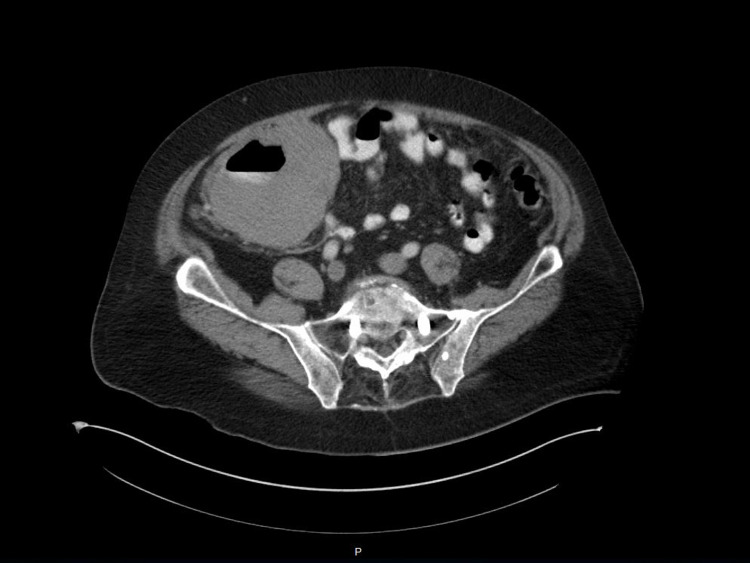
CT scan of the abdomen showing large nearly circumferential mass at the cecal and terminal ileal. CT: computed tomography

The patient was admitted to the routine medical floor for a workup of symptomatic anemia. One unit of packed red blood cell was transfused, improving his hemoglobin to 7.3 g/dL. Intravenous fluids were initiated with proton pump inhibitors. Endoscopy and colonoscopy were planned. Endoscopy was significant for severe diffuse hemorrhagic gastritis in the body of the stomach and antrum. Colonoscopy showed severe colitis that was erythematous, friable, and ulcerative in nature, which was localized in the cecum. Additionally, diverticulosis and grade II medium-sized uncomplicated external hemorrhoids were noted. Computed tomography scan showed a large nearly circumferential mass at the cecal and terminal ileal levels, measuring approximately 11.0 × 9.8 cm axially, with associated moderate luminal narrowing of the sacrum and scattered high-grade luminal narrowing of the terminal ileum, without any frank obstruction (Figure 1). There were scattered abnormal, enlarged right lower quadrant lymph nodes which measured up to 5.2 × 4.8 cm. Moreover, some scattered right lower quadrant fat stranding was noted, which was compatible with a malignancy. At this stage, surgery was consulted along with hematology/oncology.

An ex-lap and right hemicolectomy were performed. The biopsy sample was positive for mantle cell lymphoma. Fluorescence in situ hybridization (FISH) was positive for t(11;14). The patient is currently awaiting a positron emission tomography scan as an outpatient. He refused chemotherapy to pursue further care involving experimental treatments with lymphoma specialists.

## Discussion

Mantle cell lymphoma is categorized as a type of NHL, comprising approximately 2.5-6% of NHL cases [3,4]. Its cells are known to express on their surface immunoglobulins, CD5, CD19, and CD22 but not CD3, CD10, and CD23. Moreover, FISH reveals translocation t (11;14). Despite having an unfavorable prognosis, the Mantle Cell Lymphoma International Prognostic Index (MIPI) score categorizes patients into low, intermediate, and high-risk groups. The low-risk group shows a five-year overall survival rate of 60%, the intermediate-risk group a median of 51 months, and the high-risk group a median of 29 months [5]. Biomarkers are often utilized to obtain these prognostic scores such as ki-67 and SOX-11 [6,7].

In our case, the patient chose to have these markers evaluated in an external facility to pursue newly available treatments which would be discussed later. Novel therapies have evolved with bortezomib, a proteasome inhibitor. Studies have also shown promising results in combination with traditional therapies such as rituximab plus cyclophosphamide, doxorubicin, vincristine, and prednisone (R-CHOP) [8]. Others include ibrutinib, a Bruton’s tyrosine kinase inhibitor [9]. Chimeric antigen receptor-engineered T-cell (CART) has shown positive outcomes regarding the response time of refractory/relapsed mantle cell lymphoma [10,11]. Bi-specific T-cell engager transiently engages CD3+ T-cells with B-cells causing a T-cell-mediated B-cell death [12]. However, challenges still exist owing to the pathophysiological variety, difficulty in early diagnosis, high rate of progression, and recurrence.

## Conclusions

Although mantle cell lymphoma is a rare form of a gastrointestinal intraluminal lesion and difficult to diagnose preoperatively, it should be on the list of differential diagnoses. However, despite recent advancements, it still carries a poor prognosis and is treated with a combination of chemoimmunotherapy at diagnosis.
